# Preventing malaria transmission by indoor residual spraying in Malawi: grappling with the challenge of uncertain sustainability

**DOI:** 10.1186/s12936-015-0759-3

**Published:** 2015-06-24

**Authors:** Emmanuel Chanda, Themba Mzilahowa, John Chipwanya, Shadreck Mulenga, Doreen Ali, Peter Troell, Wilfred Dodoli, John M Govere, John Gimnig

**Affiliations:** Malaria Vector Control Consultant, Lusaka, Zambia; Malaria Alert Centre, Chichiri, Blantyre, Malawi; Ministry of Health, National Malaria Control Programme, Lilongwe, Malawi; USAID/PMI, Country Office, Lilongwe, Malawi; World Health Organization, Country Office, Lilongwe, Malawi; Malaria Vector Control Consultant, Nelspruit, Mpumalanga South Africa; Centers for Disease Control and Prevention, Atlanta, GA USA

**Keywords:** Malaria vector control, Indoor residual spraying, Integrated vector management, Insecticide resistance management, Vector surveillance, Sustainability, Malawi

## Abstract

**Background:**

In the past decade, there has been rapid scale-up of insecticide-based malaria vector control in the context of integrated vector management (IVM) according to World Health Organization recommendations. Endemic countries have deployed indoor residual spraying (IRS) and long-lasting insecticidal nets as hallmark vector control interventions. This paper discusses the successes and continued challenges and the way forward for the IRS programme in Malawi.

**Case description:**

The National Malaria Control Programme in Malawi, with its efforts to implement an integrated approach to malaria vector control, was the ‘case’ for this study. Information sources included all available data and accessible archived documentary records on IRS in Malawi. A methodical assessment of published and unpublished documents was conducted via a literature search of online electronic databases.

**Discussion:**

Malawi has implemented IRS as the main malaria transmission-reducing intervention. However, pyrethroid and carbamate resistance in malaria vectors has been detected extensively across the country and has adversely affected the IRS programme. Additionally, IRS activities have been characterized by substantial inherent logistical and technical challenges culminating into missed targets. As a consequence, programmatic IRS operations have been scaled down from seven districts in 2010 to only one district in 2014. The future of the IRS programme in Malawi is uncertain due to limited funding, high cost of alternative insecticides and technical resource challenges being experienced in the country.

**Conclusions:**

The availability of a long-lasting formulation of the organophosphate pirimiphos-methyl makes the re-introduction of IRS a possibility and may be a useful approach for the management of pyrethroid resistance. Implementing the IVM strategy, advocating for sustainable domestic funding, including developing an insecticide resistance monitoring and management plan and vector surveillance guidelines will be pivotal in steering entomologic monitoring and future vector control activities in Malawi.

## Background

Malaria remains a major contributor to worldwide disease burden and poverty. In the past decade, malaria control efforts have increased tremendously, culminating in appreciable declines in global burden of the disease [1]. Endemic countries have deployed efficacious vector control using indoor residual spraying (IRS) and long-lasting insecticidal nets (LLINs) as hallmark interventions, alongside case management with effective treatment using artemisinin-based combination therapy (ACT) guided by definitive diagnosis [2].

In Malawi, IRS for malaria vector control with Gammexane dates back to 1913 under the Sanitary Board Ordinance and Public Works Departments [3]. During the 1990s, small-scale IRS programmes were embarked upon by the private sector (Illovo Malawi Sugar Company) [3]. These efforts provided a model and enthusiasm for operational scale IRS implementation by the National Malaria Control Programme (NMCP). In 2007, with support from the US President’s Malaria Initiative (PMI), the NMCP piloted IRS in one high-transmission district, Nkhotakota, eventually scaling up to cover two full districts [4]. In 2010, based on the success of the initial pilot, the Government of Malawi/Ministry of Health (GoM/MoH) supported IRS in five additional districts [4]. The Malaria Strategic Plan (2011–2015), extended to end in 2016, proposes further scaling up of IRS to 15 districts [5]. However, high levels of pyrethroid and carbamate resistance were detected in *Anopheles funestus* in multiple sites across the country [6]. These findings necessitated a shift to more expensive, short-acting organophosphate insecticides. As a consequence, PMI suspended direct support for IRS in Malawi in 2012 [4].

The IRS programme aims to reduce malaria-related morbidity, mortality and poverty, and to contribute to the Malawi Growth and Development Strategy (MGDS) and Millennium Development Goals (MDGs) [5]. The GoM-funded IRS operations have declined to only one district in the 2013–2014 spraying season due to inadequate support by partners precipitated by the emergence and spread of insecticide resistance, lack of committed GoM financial resources and technical flaws [4]. Clearly, the IRS programme in Malawi is grappling with the challenge of sustaining operational-scale implementation. This paper highlights and discusses the successes and continued challenges, and presents opportunities, as an archetype for other countries to learn from the experience of Malawi.

## Case description

### Search strategy

The NMCP in Malawi, with its efforts to implement an integrated approach to malaria vector control, was the ‘case’ for this study. Information sources included all available data and accessible archived documentary records on IRS in Malawi. A methodical assessment of published and unpublished documents was conducted via a literature search of online electronic databases, Google Scholar [7], Pub Med [8], African Journals Online (AJOL), and World Health Organization (WHO) Library Database [9], using a combination of search terms: (1) malaria AND IRS; (2) IVM AND IRS; (3) IRS AND vector control; (4) Malawi; (1) and (4); (2) and (4); and, (3) and (4). Additional, non-peer reviewed literature was examined for information related to the subject.

### Study area

Malawi is a landlocked country bordered by Tanzania to the north, Zambia to the west and Mozambique to the east and south. The population in 2015 is projected to be 16.3 million, comprised of approximately 51% women and 19% children under 5 years of age [10]. Based on location and topography, Malawi is divided into three zones: the lakeshore zone, the highland zone and the lowland zone. Administratively, Malawi is divided into 28 districts among three regions (Northern: six districts, Central: nine districts, and Southern: 13 districts). Malawi’s diverse altitude ranges from 50 m above sea level in the lower Shire Valley to 2,600 m on the Nyika plateau in the north and above 3,000 m on the Mulanje peak in the south. This influences both the intensity and distribution of rainfall and temperature range. Mean annual temperature varies with altitude ranging from 25°C in the lower Shire Valley to 13°C on the Nyika plateau. Most of Malawi receives between 725 and >2,000 mm rainfall per annum [11]. Annual precipitation and wet season temperature determines the distribution of malaria vectors. Malawi provides a perfect climate and geography for *Anopheles* mosquitoes.

### Malaria epidemiological context

**Figure 1:**
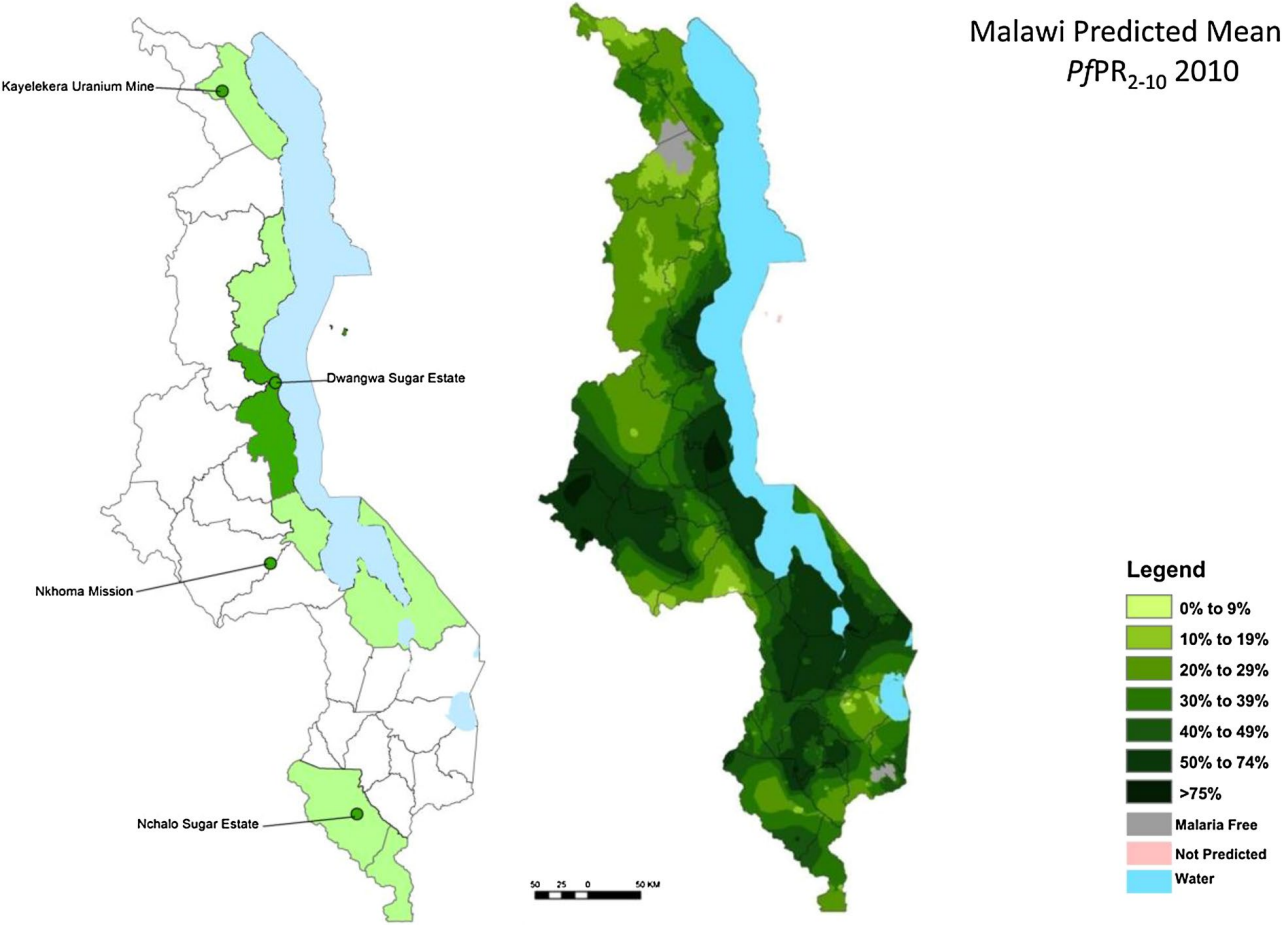
*First map* location of indoor residual spraying sites. First map: *dark green* = early adopting areas; *light green* = second phase. *Second map* Malaria prevalence. *Source* [3].

In Malawi malaria is endemic in more than 95% of the country and the entire population is considered to be at risk of the disease [11] (Figure 1). Transmission is high, defined as greater than one case per 1,000 residents and perennial with substantial seasonal variation in intensity [12]. Although *An. funestus* is the primary vector, *Anopheles gambiae s.s.* and *Anopheles arabiensis* also exist and may predominate in some areas at certain times of the year [13]. Vectorial capacity is high due to abundant rainfall (725 to >2,000 mm/annum), elevated year-round temperatures and high humidity, especially in low-lying areas along the lakeshore, Shire River Valley, and central plains [13], while the lowest risk areas fall along the highland areas of Rumphi, Mzimba, Chitipa, and Kirk Range [3, 14, 15]. Approximately 98% of malaria cases are due to *Plasmodium falciparum* and is responsible for all severe forms of the disease and deaths [3]. Attempts to control the anopheline vectors have been limited and intermittent and have had little apparent impact on the huge malaria burden [12].

### Indoor residual spraying

The NMCP is mandated to develop the overall operational design, policies and strategies and coordination and management of all vector control programmes in Malawi. IRS is the main malaria transmission reducing intervention alongside LLINs and is implemented and recorded at district level by the GoM/MoH [5]. The strategic IRS objectives include: coverage of at least 85% of all targeted structures in 12 high-transmission districts by 2015 through public, private sector and community partnerships; advocacy for the removal of taxes and tariffs of IRS commodities and supplies; and, advocacy for more resources for IRS from government and external funders [5].

### Guidelines for IRS

In 2012, Malawi developed national IRS guidelines to facilitate evidence-based, operational-scale deployment for effective vector control [16]. These guidelines are intended for use by all stakeholders implementing IRS at community, district, zonal, and national levels. The guidelines have been designed to standardize the programmatic, operational, logistical, and technical aspects necessary for implementing timely, efficient, effective, and safe IRS programmes. They also provide information on the components of the IRS programme and present technical contents, including planning, implementation, supervision, monitoring and evaluation, and reporting on IRS programmes. The IRS guidelines give the tactical direction for effective deployment and are aligned with the current WHO guidance [17] and the changing epidemiology of malaria in the country. These guidelines aim to reduce disease burden, improve the cost-effectiveness of operations and be sustainable.

### Implementation of IRS

Malawi has implemented IRS according to WHO recommendations [17] with strong adherence to country-specific guidelines [12]. Operational IRS activities range from planning and training, implementation work, monitoring and evaluation of spraying activities, environmental compliance, to entomological monitoring and surveillance. The PMI-funded Research Triangle International (RTI) supported the first IRS pilot programme by the MoH/NMCP, using lambdacyhalothrin in one district (Nkhotakota) in 2007 (Figure 1). The IRS programme aimed to ensure coverage of at least 85% of all eligible structures in targeted districts. Approximately 28,227, 42,044 and 56,729 structures were sprayed in 2007, 2008 and 2009, respectively [3]. In excess of 0.5 million people were protected between 2007 and 2009. With government support, the IRS programme using pyrethroids was scaled-up to cover a total of seven highly endemic districts along the lakeshore and the lower Shire Valley in 2010, 2011 and 2012, with 527,372 (78.6%), 653,592 (85.2%) and 575,945 (6.1%) structures sprayed accordingly [3]. The expansion was in line with the current NMSP III 2011–2015 strategic objective of scaling the intra-domiciliary spraying programme to a total of 12 districts by 2015 [5]. However, Malawi scaled back the IRS programme from seven districts in 2011 to only one district (Salima) by 2014 [4].

### Challenges to IRS implementation

#### Selection for insecticide resistance

The development and spread of pyrethroid resistance in malaria vectors, particularly *An. funestus* has been documented extensively across Malawi and has adversely affected the IRS programme [6, 18]. In 2002, *An. arabiensis* exhibited resistance to dichloro-diphenyl-trichloroethane (DDT) but remained susceptible to pyrethroids and organophosphates [18]. In 2009, pyrethroid and carbamate resistance was detected in *An. funestus* in Likoma Island, Nkhotakota and Salima districts and prompted a switch from the use of pyrethroids to a shorter residual lifespan organophosphate (pirimiphos-methyl) [19, 20]. By 2011, pyrethroid and carbamate resistance had been widely confirmed, limiting the insecticide options for IRS [6]. In Karonga district in the north, *An. arabiensis* is the main malaria vector and remains susceptible to pyrethroids. However, this species is resistant to pyrethroids in some parts of southern Malawi [4]. While full susceptibility of *An. funestus* to the organophosphate malathion and DDT was confirmed in 2012, suspected resistance to DDT in the same species has been reported in certain parts of the country [6]. Malawi scaled back the IRS programme to only one district in 2012, due to the inhibitory cost of organophosphate insecticides [4].

#### Operational and technical concerns

In addition to the emergence and expansion of pyrethroid and carbamate resistance, IRS activities in Malawi have been characterized by substantial inherent logistical and technical tribulations culminating in missed targets during the spray campaign [4]. The constraints include: (1) inadequate, unpredictable flow and late disbursement of GoM funds leading to delayed spraying from the scheduled November/December until April/May of 2014; (2) the refusals or non-compliance by residents; (3) reduction in the number of targeted districts for IRS from six to one district; (4) switching of insecticides from pyrethroids to a more expensive and short-acting organophosphate, which ultimately made the IRS programme unsustainable; (5) a lack of consensus among stakeholders on the use of DDT for IRS due to fears of contamination; and, (6) procurement of low-quality insecticides and spray pumps by the GoM/MoH. However, to establish effective vector control, Malawi has developed a stakeholder-driven and evidenced-based integrated vector management (IVM) strategy [21]. PMI committed itself to providing technical assistance to the GoM in 2012–2014 for IRS activities and will continue to support ongoing entomological and resistance monitoring [4].

#### Financial support constraints

Initially PMI was the only donor providing direct funding support for IRS in Malawi. Given the early success of the PMI-financed IRS programme, the GoM rapidly increased support for IRS in non-PMI districts. However, in 2012 PMI suspended direct funding support to GoM/MoH for IRS activities due to the increased cost (up to 15% of PMI budget) to protect just 3% of the population and funding levels from the GoM have steadily declined. Continued support could not be justified within the existing budget envelope without seriously jeopardizing other intervention areas of the PMI Malawi programme [4]. The lack of PMI funding had created uncertainty of Malawi conducting full-scale IRS operations after 2014. While *An. funestus* is still susceptible to organophosphates, the cost is ten to 15 times that of pyrethroids, which drastically increases the overall operational costs of IRS. Moreover, only a short-acting organophosphate (OP) was available, given the high cost and short duration of residual efficacy. With the already declining support for IRS, sustainability of financial support from the GoM for IRS remains uncertain and has grave implications for malaria control in the country. In the event that PMI reconsiders IRS in Malawi, the likelihood that it will again be a solitary partner is high.

### Opportunities for strengthening IRS

#### Improving vector surveillance and insecticide resistance management

Malawi does not presently have national guidelines to facilitated well-coordinated malaria vector surveillance. Mapping of vector species and their resistance profiles across the country would facilitate deployment of cost-effective vector control. As such, vector surveillance should be an integral aspect of the IVM strategy implementation to: (1) provide evidence for decision-making in IRS; (2) evaluate the programme’s impact on vector populations; and, (3) monitor and evaluate IRS where the surveillance sites are located in or near implementation settings [22]. Malawi intends to develop and establish country-specific vector surveillance system to conduct regular entomological investigations at fixed locations to: (1) reduce natural variation, costs and labour intensity; (2) increase the usefulness of timely collected data in decision-making; and, (3) optimize the use of available resources. It is envisioned to rotate districts that are monitored with up to seven districts under monitoring each year. Insecticide resistance data show that organophosphates and possibly DDT are the only technically sound options for IRS in Malawi. However, presently there is no operational insecticide resistance management (IRM) plan in the country. A rational IRM approach is required to guide evidence-based decisions regarding insecticide choices for IRS. Switching to strategic IRM demands for meticulous situation analysis and development of national insecticide resistance monitoring and management plans according to the WHO recommended framework [23]. Continued PMI support for entomologic monitoring, including any districts where IRS is conducted, provides an opportunity for effective IRS deployment as outlined in the IVM strategy.

#### Strengthening mapping and IRS data collection and reporting

Improving geographical reconnaissance (GR) supported by GIS-based satellite imagery is critical for Malawi to enumerate each targeted household and will improve IRS planning, operations, logistics, advocacy, and monitoring. Moreover, the approach is cheaper, faster, requires fewer human and financial resources and ensures 100% coverage of targeted households with satellite view. This will also facilitate targeting and prioritization of eligible spray areas together with operations and real-time monitoring of spray coverage. The IRS database for Malawi needs to be updated by establishing a national IRS information/data collection and reporting tool in line with the WHO standard format for IRS. The data to be recorded include IRS policy/strategy outlining the objective for spraying (malaria elimination, seasonal or perennial malaria control, epidemic response) and specify whether targeted or blanket spraying, and the database should indicate type of insecticides and quantities used. It must include IRS implementation, supervision and quality monitoring using cone bioassay, including vector surveillance. The tool should also incorporate current pesticide management capacity of the IRS programme.

#### Consolidating collaboration, coordination and information, education and behaviour change communication

The MoH/NMCP, in conjunction with other stakeholders in Malawi, developed an evidence-based IVM strategy founded on thorough situation analysis of vector control tools, entomological and insecticide resistance, and epidemiological evidence [21]. The IVM strategy is important in forging strong partnerships and providing the tactical direction for effective deployment of vector control interventions along the five key elements of the approach and to align them with changing epidemiology of the disease in the country. It outlines IRS and LLINs as key strategic interventions including larviciding using *Bacillus thuringiensis* var *israelensis* and environmental management as alternative tools where feasible, IRM, monitoring and evaluation and operational research, programme management, budget and funding, and an implementation plan. The IVM strategy is expected to reduce the risk of transmission, reduce disease burden, improve the cost-effectiveness of vector control operations, improve ecological soundness and be sustainable [21]. In this regard, resources for IRS could be mobilized by strengthening advocacy and collaboration based on the IVM strategy. Informing, educating and community mobilization, at all stages of the spraying cycle, has been an integral component of the IRS programme in Malawi [24]. This requires unremitting, well-coordinated and harmonized information, education and behaviour change communication (IEC/BCC) to promote knowledge, awareness and compliance and ownership of IRS. Equally, strategic and effective advocacy for increased and continued political, financial and technical support and to mobilize all stakeholders (local public and private) support will be vital for the sustainability of the IRS programme.

## Discussion

The IVM policy has been adopted and implemented as the main approach to vector control by most WHO member states [25]. Well-established IVM programmes with adherence to the five key attributes of the approach have demonstrated enhanced impact of interventions and opened a window for leveraging additional resources [26]. In Malawi, the malaria programme review, undertaken in 2010, informed the development a Malaria Vector Control Strategy (2015–2019) in 2014. The strategy was founded on the principals of IVM and spell out tactics and recommendations for improving vector control in Malawi [21]. The development process involved engagement of research organizations and implementing partners to: (a) assess the operational impact of pyrethroid resistance; (b) explore the potential use of DDT and other long-lasting insecticide formulations for IRS; and, (c) explore the role of alternative vector control innovations in the context of IVM. The strategy prioritizes: (1) use of IRS with non-pyrethroid and non-carbamate insecticides in highly malaria-endemic areas based on epidemiological (along the lakeshore and lower Shire Valley) and entomological (insecticide resistance) data; (2) where feasible, supplement IRS and LLINs with focal larval source management (LSM), preferably using bio-larvicides, according to WHO guidelines; and, (3) determining the diversity of vectors and establishing rational IRM strategies (rotational or mosaic approaches) [21].

Compromised management of insecticides in agriculture and in public health can lead to selection of insecticide resistance in disease vectors and undermine vector control [6]. Consolidating the strategic frameworks for IVM will strengthen the IRS efforts, improve management of insecticides and environmental safeguards, and facilitate management of insecticide resistance. It will enhance intersectoral accountability, leading to responsible actions among a wide range of stakeholders and provide a platform for sustaining and maximizing the impact of IRS in a cost-effective manner [11]. As such, the NMCP should use and manage pesticides judiciously in the context of IVM. High political obligation to combating communicable diseases exists in Malawi. This is exemplified by the availability of a national health strategic plan and good collaboration with various partners, coupled with the commitment of relevant government ministries to jointly support vector control. To ensure adequate stakeholder participation in the planning, design and deployment of interventions, provision of guidance and technical insight to policymakers, improving IEC/BCC and advocacy are necessary.

Malawi is among the countries with the highest malaria transmission intensity worldwide [12]. In order to successfully control malaria and move towards elimination, the country needs to learn from the experience of other countries in scaling-up proven high-impact vector control interventions. Using the platform of the IVM strategy, some endemic countries have propped up vector control by placing it high on the political agenda, and are making steady progress towards focalized elimination of malaria [27]. LLINs and IRS are deployed as main thrust interventions supplemented with LSM in accordance with and strict adherence to a set of eligibility criteria [28, 29]. Botswana, Eritrea, Namibia, South Africa, and Swaziland have implemented and sustained IRS with consistent national government funding supplemented by partner support [29, 30]. This has resulted in marked reduction in malaria transmission and these countries are now re-orienting toward malaria elimination [31, 32]. In Zambia, IVM has facilitated strong partner collaboration and has helped leveraging of additional resources for IRS [27]. Over the years, IRS operations have been financed by the Global Fund, World Bank, PMI, and recently Malaria Control and Evaluation Programme (MACEPA), alongside Zambian Government funding [33]. Zambia has strengthened GR by incorporating GIS-based satellite imagery to improve IRS planning, targeting, operations, logistics, advocacy and monitoring, and evaluation [34]. Namibia and Eritrea have recently developed costed IVM strategies for advocacy and leveraging of resources for evidence-based vector control [35, 36].

Strong entomological teams at national and local levels are crucial to coordinate routine monitoring of resistance, data analysis and interpretation to inform policy decisions, translate policies and guidance into action at ground level. Zambia, Eritrea and Namibia have developed country-specific IRM plans to prevent development and spread of insecticide resistance and have trained local staff in entomological and resistance monitoring [31]. Zambia and Zimbabwe have also established strong external linkages with international research institutions to further build local entomological capacity. Namibia has elaborated vector surveillance guidelines to facilitate entomological monitoring by the regional levels and have streamlined reporting tools for DDT [35]. Both Eritrea and Namibia have been conducting contact bioassays and insecticide resistance monitoring over the years and have standardized their IRS data collection and reporting tools in line with WHO guidance. Strengthening environmental safeguards through collaboration with in-country environmental regulatory bodies is also necessary for efficient use of insecticides including DDT.

## Conclusions

IRS is a proven and effective malaria vector intervention if correctly implemented using WHO recommended insecticides. However, the future of the IRS programme in Malawi is uncertain due to limited funding, cost of alternative insecticides and technical resource challenges being experienced in the country. DDT is currently not registered for public health use in Malawi due to environmental concerns and strong opposition from the agricultural sector over potential contamination of crops, mainly tobacco which is the main foreign exchange earner, and the inherent loss of export markets. However, the availability of a long-lasting formulation of pirimiphos-methyl makes the re-introduction of IRS a possibility and may be a useful approach for the management of pyrethroid resistance. While the GoM is prioritizing the execution of its malaria control activities, it remains unknown if IRS will remain a high-priority intervention. Future decisions on whether and where to implement IRS will be guided by the IVM strategy. Therefore, implementing the IVM strategy, advocating for sustainable domestic funding and developing an IRM plan and a vector surveillance guideline will be critical in steering entomologic monitoring and future vector control activities in Malawi.

## Abbreviations

ACT: artemisinin-based combination therapy
BCC: behaviour change communication
DDT: dichloro-diphenyl-trichloroethane
GoM: Government of Malawi
GPIRM: global plan for insecticide resistance management
IEC: information, education and communication
IRS: indoor residual spraying
IRM: insecticide resistance management
IRMTWG: Insecticide Resistance Management Technical Working Group
LLINs: long-lasting insecticidal nets
LSM: larval source management
NMCP: National Malaria Control Programme
WHO: World Health Organization
